# The Emerging Role of Insulin and Insulin-Like Growth Factor Signaling in Cancer Stem Cells

**DOI:** 10.3389/fendo.2014.00010

**Published:** 2014-02-04

**Authors:** Roberta Malaguarnera, Antonino Belfiore

**Affiliations:** ^1^Endocrinology, Department of Health Sciences, University Magna Graecia of Catanzaro, Catanzaro, Italy

**Keywords:** IGF-I, IGF-IR, IGF-II, insulin, insulin receptor, stem cells, cancer stem cells, EMT

## Abstract

Cancer cells frequently exploit the IGF signaling, a fundamental pathway mediating development, cell growth, and survival. As a consequence, several components of the IGF signaling are deregulated in cancer and sustain cancer progression. However, specific targeting of IGF-IR in humans has resulted efficacious only in small subsets of cancers, making researches wondering whether IGF system targeting is still worth pursuing in the clinical setting. Although no definite answer is yet available, it has become increasingly clear that other components of the IGF signaling pathway, such as IR-A, may substitute for the lack of IGF-IR, and induce cancer resistance and/or clonal selection. Moreover, accumulating evidence now indicates that IGF signaling is a central player in the induction/maintenance of epithelial mesenchymal transition (EMT) and cell stemness, two strictly related programs, which play a key role in metastatic spread and resistance to cancer treatments. Here we review the evidences indicating that IGF signaling enhances the expression of transcription factors implicated in the EMT program and has extensive cross-talk with specific pathways involved in cell pluripotency and stemness maintenance. In turn, EMT and cell stemness activate positive feed-back mechanisms causing up-regulation of various IGF signaling components. These findings may have novel translational implications.

## Introduction

The IGF system exerts a fundamental role in the regulation of growth, development, and metabolism in response to nutrients. However, several lines of evidence have now well established that this complex signaling network is also exploited by cancer cells to sustain their growth and resistance to apoptosis. The IGF system comprises two homologous receptors, the insulin and the IGF-I receptor (IR and IGF-IR) and a third unrelated receptor, the mannose 6-phosphate receptor (M6P/IGF-IIR), four secreted molecules (insulin, IGF-I, IGF-II, proinsulin) characterized by various binding affinity to these receptors, and six binding proteins (IGF-BPs) (1, 2). Differential splicing of IR gene produces two IR isoforms that differ for the exclusion (IR-A) or the inclusion (IR-B) of a small exon (exon 11) that encodes a stretch of 12 aminoacids located at the carboxyterminus of the IR α-subunit. These two isoforms bind insulin with similar affinity, but differ mainly for their binding affinity to IGF-II and proinsulin, which is high for IR-A, but very low for IR-B (3, 4). Upon ligand binding, IR and/or IGF-IR become phosphorylated on several tyrosine residues coupled to specific intracellular signaling pathways, including the Ras/Raf/MAPK and the PI3K signaling cascades. In contrast, the M6P/IGF-IIR binds only IGF-II, and targets it to lysosomal degradation, thus counteracting IGF-II mitogenic effects (5).

Indeed, most malignancies show IR and IGF-IR overexpression together with loss of M6P/IGF-IIR, increased expression of autocrine and/or paracrine IGF-I and IGF-II, and dysregulated IGF-BPs production (6, 7). In particular, IR-A overexpression and increased IGF-II autocrine production have been recognized to have a key role in the progression of several malignancies. The IGF system and its signaling in physiology and disease have been recently reviewed by several papers to which we refer for more detailed information (3, 6, 8).

Because of the frequent dysregulation of the IGF system in cancer, various components of this system have recently become attractive targets for anticancer therapies. One of the main approaches pursued has been IGF-IR targeting, on the basis of IGF-IR prominent involvement in growth regulation and its scarce contribution to glucose metabolism (9). Instead, targeting the IR has been considered a less desirable option because of its major involvement in glucose metabolism, and the potential derangement of glucose metabolism arising from IR blockade. However, a few clinical trials using IGF-IR blocking antibodies have recently failed our expectations, as only a small number of malignancies (Ewing’s sarcomas and a small subset of NSCLC lung tumors) have shown significant objective response to these treatments (10).

New insights useful for targeting the IGF system may come from evidences indicating that this system is involved in stem cell biology, and that IGF system dysregulation may contribute substantially to the growth/expansion of cancer stem-like cells. In fact, accumulating evidence strongly suggests that malignant tumors are driven and sustained by a subset of non-differentiated cells with stem cell-like properties, such as self-renewal, tumorigenicity, and multi-lineage differentiation abilities (11). According to this model of tumorigenesis, the early step in tumor development is the clonal expansion of initiating stem cell pool through the unbalance between self-renewal and differentiation capacities. For this reason, it has been suggested that these cells and/or their immediate progeny may be targets for transformation (12).

The most clinically relevant features of cancer stem cells (CSCs) are the inherent radio/chemo-resistance and the capacity to metastasize (13). Recent studies have linked these features with the epithelial-to-mesenchymal transition (EMT), a process in which adherent epithelial cells loose their epithelial characteristics and acquire mesenchymal properties, including fibroblastoid morphology, invasive and migratory potential, self-renewal capacity, and specific gene-expression changes similar to those of stem cells (14). Herein, we will review recent studies that have provided increasing evidence that IGF signaling is crucial in the maintenance of stem-like phenotype of cancer cells by contributing to regulate EMT, pluripotency, and self-renewal. This emerging concept will undoubtedly stimulate renewed efforts aimed at co-targeting the IGF system and other pathways that contribute to CSCs biology.

## IGF Signaling in Normal and Cancer Stem Cell Biology

Nowadays, there is strong evidence in support of a role for IR/IGF-IR dependent signals in the biology of normal progenitor/stem cells (15–20). In fact, it has been shown that self-renewal, pluripotency, survival, and expansion of human embryonic stem cells require IGF-II produced by the supportive niche (15). Interestingly, neural progenitor/stem cells have been found to express both IGF-IR and IR-A, with a predominance of the latter, and to be exquisitely sensitive to IGF-II for self-renewal. In contrast, lineage restricted progenitors expressed preferentially the IGF-IR and responded to IGF-I (21). An IGF-II analog that binds the IR-A but not the M6P/IGF-IIR was still able to promote self-renewal of neural stem cells also in the presence of blocking antibodies against the IGF-IR, further reinforcing the concept that IGF-II promotes stemness through the IR-A, and not through the M6P/IGF-IIR or the IGF-IR (22).

Moreover, an important role of the IGF system has been recognized in cancer progenitor/stem cells from solid and hematopoietic malignancies. In this context, we have found that IR and IGF-IR, as well as cognate ligands IGF-I and IGF-II, are overexpressed in human thyroid progenitor/stem cells cultured as thyrospheres. IR-A and IGF-II were especially predominant in thyrospheres from cancer cells, where only IGF-II stimulated self-renewal ability. However, both IGF-I and IGF-II were able to stimulate sphere volume. IR-A and IGF-IR, as well as cognate ligands markedly declined in differentiating cells (23). Similar findings have been reported also in hepatocellular carcinoma, where IGF-IR and IGF-II appear to be implicated in self-renewal ability of hepatic CSCs (24).

Recently, it has been shown that IGF-IR expression and activation is greater in breast CSCs, as compared to normal breast stem cells (25). IGF-IR knockdown or the specific inhibition of its downstream signaling, was able to reduce the pool of breast CSCs, their capacity to undergo the EMT process and to form mammospheres *in vitro* and tumors *in vivo*. These data indicate that, in breast cancer cells, IGF-IR may be considered as a marker of stemness and a suitable therapeutic target. Similarly, high expression of IGF-IR in lung adenocarcinoma cells was positively correlated with the expression of CSC markers, while IGF-IR blockade inhibited cell self-renewal and tumorigenicity *in vivo* (26).

Yet, chemoresistant colorectal cancer cells with CSC phenotype, showed over-activated IGF-IR signaling, which was responsible for enhanced sensitivity to IGF-IR-targeted therapy (27). Actually, IGF-IR signaling appeared to enrich chemoresistant populations of colon CSCs, which are selectively sensitive to the anti-IGF-IR antibody figitumumab (28). In hepatocellular carcinoma cells, resistance to the EGFR inhibitor gefitinib and cell stemness markers were found associated with increased IGF-IR nuclear translocation (29), a phenomenon recently described to occur in cancer cells (30). Although the functional role of IGF-IR nuclear translocation is still unclear, it seems to be involved in transcription enhancement of certain genes, among which the IGF-IR gene itself, thus promoting a feed-forward loop (31).

Recently, two groups have demonstrated that IGF-IR mediates important growth/survival signals also in hematopoietic tumors. Medyouf et al. reported that moderate levels of IGF-IR signaling are sufficient for the expansion of bulk lymphoblastic leukemia cell population (32). However, high levels of IGF-IR, and activation of its downstream signals are required for sustained growth of human T acute lymphoblastic leukemia stem cells. Most intriguing, the same authors have shown that moderate inhibition of IGF-IR signaling compromises leukemia-initiating cell activity and their transplantability in syngeneic/congenic secondary recipients. Others have shown that IGF-IR signaling contributes to the malignant transformation of normal committed myeloid progenitors, which were responsive to the growth inhibitory effect of IGF-IR/IR selective tyrosine kinase inhibitors (33).

In summary, a growing body of evidence highlights the importance of IR-A and IGF-IR in regulating stem cell biology, and supports the notion that IGF-I levels in the newborn are positively related to the total number of stem cells, which is associated with the risk of future cancers (19). These studies also raise the possibility to explore IGF-IR specific pharmacological inhibition for a variety of human malignancies, in order to sensitize chemoresistant stem-like cells to classical therapies and reduce relapse rates.

## A Complex Role of IGF Signaling in EMT Program

### The EMT program and cancer stem cells

The EMT program regulates the transition of epithelial cancer cells from an epithelial to a motile mesenchymal morphology. EMT has an important role during embryogenesis and is highly conserved in adult life to guarantee plasticity, tissue repair, and maintenance. A key feature of EMT is the decreased expression of E-cadherin, a transmembrane cell adhesion molecule important in maintaining epithelial cellular polarity, along with increased expression of mesenchymal markers such as vimentin, fibronectin, and N-cadherin. These changes drive the transformation of adhesive, non-motile epithelial-like cells into motile, stem-like cells.

Although the origin of CSCs is controversial, recent observations support the notion that they may arise by the activation of the EMT program, which is the first step for tumor metastatic dissemination. Indeed, by either overexpressing EMT-inducing transcription factors, Snail or Twist, or by exposing the cells to TGFβ, it is possible to induce stemness features in non-tumorigenic human mammary embryonic cells, as well as to increase tumorigenicity in xenotransplants (14, 34). Furthermore, high expression of EMT markers in colorectal, breast, and ovarian cancers correlates with the de-repression of stemness gene signature, the development of chemo-/radio-resistance, and the acquisition of metastatic potential (34–36). These evidences strongly suggest that the EMT process has a close relationship with CSCs and their metastatic ability. A complex network of factors that includes growth factors, cytokines, transcription factors, and the tumor microenvironment tightly regulates the EMT process. The transcription factors controlling EMT program belong to the ZEB, Snail, and Twist families. These factors are tightly regulated at transcriptional, translational, and epigenetic level, and act as molecular switches favoring the induction of EMT. They recognize the E-box DNA sequences in the promoter region of E-cadherin, recruit cofactors and the histone deacetylase, and specific miRNAs, causing down-regulation of E-cadherin expression. Deregulation of EMT-activating transcription factors have been observed in several cancers. Snail has been linked with tumor grade, metastasis, recurrence, and poor prognosis (37). Snail and Twist further cooperate in inducing the expression of ZEB1, which controls genes relevant in metastasis and migration of CSCs also by repressing stemness-inhibiting microRNAs (38). For their key role in endowing cancer cells with stem-like properties and with aggressive pro-invasive phenotype, these transcription factors have been suggested to be not only attractive diagnostic and prognostic biomarkers but also potential therapeutic targets.

### IGF signaling and EMT program: A strict relationship

Stimulation of the IGF axis in immortalized or in cancer cells is now recognized to be able to induce up-regulation of several transcription repressors involved in the EMT process. More than one decade ago, IGF-I was shown to increase mRNA and protein expression of Twist, not only in mouse fibroblasts overexpressing the human IGF-IR, but also in mouse skeletal muscle, through the activation of the MAPK pathway (39). Although this study was carried out in non-transformed mesenchymal mouse cells, it is noteworthy that IGF-I antiapoptotic effect was partially mediated by Twist up-regulation.

Several studies have been conducted in malignant or immortalized epithelia cells. The MEK/ERK pathway was found also involved in ZEB1 up-regulation by IGF-I (40). In ARCaPE prostate cancer cells, which have an epithelial phenotype and low ZEB1 expression, IGF-I is expressed and secreted at low levels. When cells were exposed to IGF-I, they showed increased ZEB1, N-cadherin, and fibronectin, resembling the more mesenchymal cell variant ARCaPM. ZEB1 blockade up-regulated E-cadherins and suppressed cell motility and invasive potential (40). Although ZEB1 activation was downstream the MEK/ERK pathway, inhibition of this pathway was only partially able to revert the epithelial phenotype, possibly because of the induction of irreversible changes.

In addition to the MEK/ERK pathway, which is implicated in Twist and ZEB1 up-regulation, GSK3β is now recognized as an essential EMT regulator in response to IGFs. Initially identified as an enzyme involved in the regulation of glycogen synthesis in response to insulin, GSK3β is known to play a key role in the canonical Wnt signaling by phosphorylating β-catenin and inducing its proteasomal targeting and degradation (see below) (41, 42). In addition, GSK3β also regulates EMT in a more complex way, which involves direct reduction of Snail and Slug expression (43). With regard to Snail, GSK3β is able to phosphorylate it, causing proteasome dependent degradation (44). GSK3β also represses Snail transcription via NF-kB activation (45). In lung cancer, it was shown that GSK3β also phosphorylates Slug protein, causing its ubiquitination by the E3 ligase, carboxyl terminus of Hsc70-interacting protein (CHIP), for proteasomal degradation (46). Inhibition of Akt reversed this phenotype, while inhibition of GSK3β contributed to the mesenchymal phenotype. Accordingly, Slug is required for metastatic spread of the transformed melanoma cells (43), and GSK3β inhibition was associated with increased Slug and N-cadherins expression and prevented melanoma cells interaction with stromal cells and migration (47). Normal epithelial cells have high basal GSK3β activity (48) and express, therefore, low levels of Snail and Slug as a result of decreased transcription and/or accelerated degradation of these factors.

Both in normal and cancer cells, GSK3β inactivation, through Ser9 phosphorylation, may occur by stimulation of the PI3K/Akt and the MAPK pathways in response to several mitogens, including insulin and paracrine/autocrine IGFs. However, in certain cancers GSK3β may be also inactivated by the occurrence of molecular abnormalities, including the expression of constitutively activated Akt3 and PTEN inactivation (49, 50). Several studies addressing the relationship between IGF signaling and EMT have been carried out in mammary cells. Early studies have shown that breast cancer cells overexpressing IGF-IR acquire depolarization and EMT phenotype following IGF-I stimulation (51). However, also in non-transformed human breast cells MCF-10A, overexpression of a constitutively active IGF-IR induced EMT phenotype, anchorage-independent growth, invasion, and tumorigenesis *in vivo*. Snail was markedly up-regulated, while no changes were observed with regard to Slug, Twist, or ZEB1. Snail up-regulation was dependent on NF-kB activation downstream IGF-IR. In fact, all these changes were reverted by either NF-kB or IGF-IR blockade. Other studies have indicated that GSK3β inhibition by Akt induces Snail and ZEB1/2 transcription via NF-kB (45, 52, 53), and that NF-kB binds Snail promoter and increases its activity (54). In addition, Akt induced NF-kB activation increases Snail stability (55). IGF-I dependent induction of EMT phenotype in MCF-10A cells required specific down-regulation of Akt1 together with maintained Akt2 expression and increased ERK/MAPK signaling (56). These studies highlight Akt isoform-specific effects on EMT and migration in breast cells.

The insulin receptor substrate (IRS) proteins, which are key effectors of both IR and IGF-IR, have also been involved in tumorigenesis and in disruption of the normal epithelial phenotype in mammary epithelial cells (57). In agreement with these findings, it has been shown that MEMO1 (mediator of ErbB2-driven cell motility 1) interacts with IRS-1 in a phospho-Tyr-dependent manner and prevents IRS-1 dephosphorylation (58). As a consequence of IRS-1 increased signaling, MCF-10A-IRS-1 and MCF-10A-MEMO1 cells, showed cell scattering and loss of cell–cell contacts, together with increase of N-cadherin and vimentin expression, and down-regulation of the epithelial markers E-cadherin, occludin, and β-catenin.

Interestingly, EMT may in turn trigger autocrine IGF-I production, which activates a positive feed-back loop between IGF-IR activation and SLUG expression, as shown in an *in vitro* model of SLUG-induced EMT based on MCDK kidney fibroblasts (59). Moreover, Snail suppresses PTEN expression, thus potentiating the PI3K/Akt pathway (60).

**Figure 1:**
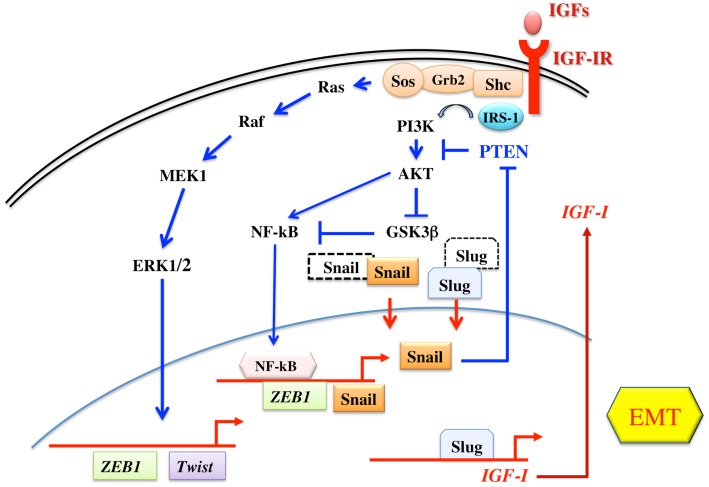
**Schematic representation of IGF signaling involvement in EMT**. The two main signaling pathways downstream the IGF-IR are both involved in the regulation of transcription factors of the Twist, Snail, and ZEB1 families, all involved in the EMT program. The IRS-1/PI3K/Akt pathway leads to the phosphorylation and inactivation of GSK3β, thus preventing the phosphorylation and proteasome targeting of Snail and Slug. The same pathway also induces the activation of NF-kB transcription factor, which then increases the transcription of Snail and ZEB1. On the other hand, the Ras/Raf/ERK pathway is involved in the transcription of Twist and ZEB1. In turn, Snail inhibits PTEN gene transcription, thus potentiating the IRS-1/PI3K/Akt pathway. In addition, Slug increases the transcription of IGF-I, the main IGF-IR ligand.

Taken together, these findings indicate that the main IGF-IR downstream pathways, the MAPK and the IRS/Akt/GSK3β cascades, are potent inducers/activators of the transcription repressors involved in EMT process. In turn, EMT process induces a positive feed-back mechanism leading to increased IGF-IR signaling through autocrine IGF-I production and reduced PTEN activity. Figure 1 shows the relationship between the IGF system and the EMT process.

### IR-A overexpression, both a consequence and a cause of EMT

IR-A overexpression coupled with IGF-II autocrine production may play an even more important role than IGF-IR overexpression in mediating EMT. IR-A expression is physiologically enhanced during prenatal life, and plays a major role to maximize IGF-II and proinsulin biological effects (4, 61). In hepatoblastoma cell lines, IR-A and IGF-II expression were positively correlated with EMT and predicted sensitivity to the dual IGF-IR – IR inhibitor OSI-906 (62). These data are reminiscent of similar data obtained in thyroid cancer cells (63), showing that anaplastic cancer cells, characterized by an EMT phenotype, express markedly elevated levels of both IR-A and IGF-II, as compared with more differentiated, papillary or follicular, thyroid cancer histotypes. Accordingly, overactivation of the IR pathway has been recognized as a relevant mechanism involved in resistance to IGF-IR blockade. In fact, resistant Ewing’ sarcomas or breast cancers may show IR-A overexpression and enhanced activation by autocrine IGF-II production (64, 65).

Several data suggest that IR-A favors a less differentiated phenotype while IR-B has the opposite effect. For instance, IR-A transfection in HepG2 hepatoblastoma cells increased cell proliferation and migration in response to IGFs and insulin, while transfection with IR-B isoform decreased both biological effects (66). Similar data were observed in colon cancer cells, where transfection with IR-B decreased cell proliferation and increased biomarkers of differentiation (67). In 32D cells transfection with IR-B, but not with IR-A, was able to favor differentiation as measured by myeloperoxidase expression (68). Additionally, mouse intestinal epithelial stem cells and progenitors are characterized by high IR-A:IR-B ratio (67). Noteworthy, activation of the insulin receptor contributes to downregulate PTEN, representing an additional mechanism of PI3K pathway activation (69).

Conversely, induction of differentiation in progenitor/stem cells from human thyroid was associated with a dramatic decrease in both absolute IR-A content and in IR-A:IR-B ratio, as well as in reduction of autocrine IGF-II (23). HepG2 cells may also be induced to differentiate and to assume a more typical epithelial phenotype. These changes are associated with a marked decrease of IR-A:IR-B ratio from 80:20 to 20:80, confirming that a bidirectional association exists between high IR-A:IR-B ratio and loss of epithelial differentiation and EMT changes (66, 70).

These findings are highly consistent with the concept that, although transcriptional regulation is required in driving EMT, a coordinate program of alternative splicing is also important (71). In fact, the newly identified splicing factors ESRP1 and ESRP2 have been also shown to be downregulated by transcription repressors Twist, Snail, and ZEB (71). Additionally, complex alterations in various splicing factors may mediate the increased IR-A:IR-B ratio during EMT. Although incompletely known, these alterations may involve increased hnRNP A1 expression and reduced hnRNP F and SF2/ASF (72). Interestingly, activation of the EGFR/ERK pathway was associated with increased IR-A:IR-B and dysregulation of several splicing factors, including CUGBP1, hnRNPH, hnRNPA1, hnRNPA2B1, and SF2/ASF, involved in IR gene splicing (73).

## Cross-Talk of IGF Signaling with Transcription Factors and Pathways Involved in Normal and Cancer Stem Cell Biology

From the seminal paper of Takahashi et al. (74), we have learnt that adult dermal fibroblasts could be reprogramed to acquire pluripotency, a key property of stem cells, with a combination of only four transcription factors (Oct-3/4, SOX2, Klf-4, and c-Myc). An additional transcription factor, Nanog was required in the final stages of cell reprograming. However, there is some flexibility in transcription factors required for cell reprograming. For instance, Myc is not absolutely required, and a different transcription factor combination (Oct-4, SOX2, Lin28, and Nanog) can work equally well (75). Cell reprograming is more efficient when p53 is inactivated (76), indicating that p53 plays also a key role in the regulation of stemness. For instance, p53 directly down-regulates pluripotency genes, such as Oct-4 and Nanog (77, 78). In turn, Nanog, by upregulating FAK, a negative regulator of p53 transcriptional activity, represses p53 expression (79). In accordance with these findings, p53 mutant forms have been found to induce EMT activators (ZEB1, ZEB2, Slug, and Snail) and stem cell expansion (80, 81).

Moreover, stem cell ability to self-renew and differentiate into specialized lineages is regulated by microenvironmental signals present in the stem cell niche. These signals include those belonging to the pathways of Wnt, Notch, Sonic hedgehog (Shh), SAT3/5, TBFβ, and others, which acts individually or by integrating with other signals (82, 83). Several of these pathways are frequently dysregulated in cancer and may play crucial roles in stem-like cancer cells.

Intriguingly, the IGF system is linked to these transcription factors and signaling pathways by a complex network of interactions.

### Cross-talk with pluripotency transcription factors

One link between IGF signaling and pluripotency factors is represented by p53, which is a common negative regulator of IGF-IR (see next paragraph), and of Oct-4 and Nanog (78). In turn, IGF-IR signaling induces p53 phosphorylation and inactivation (84), thus relieving p53 suppression on Oct-4 and Nanog. Another link is represented by hypoxia factors 1 and 2 (HIF-1 and HIF-2), which orchestrate the up-regulation of several factors involved in cell transformation, EMT, and pluripotency, including Twist, Snail, Oct-4, SOX2, and Nanog (85). Besides being activated by hypoxia, HIFs are also activated by several growth factors, including IGFs (86) through the PI3K/Akt/mTOR pathway (87).

Moreover, IGF-IR may modulate the expression and function of pluripotency factors through the cross-talk with the Wnt/β-catenin pathway (see Section Cross-Talk with Wnt/β-Catenin Signaling). In particular, it has been shown that the IGF-IR/PI3K/GSK3β pathway mediates Oct-4 up-regulation as well as the formation of β-catenin/Oct-4/SOX2 complex, which is able to activate the Nanog promoter and maintain self-renewal of lung cancer stem-like cells (26). These data fit well with findings obtained in human hepatocarcinoma. In this malignancy, Nanog expression correlates with poor prognosis. Moreover, Nanog+ cell populations showed the hallmarks of stemness, which were dependent on the presence of a functional IGF-IR. Nanog+ cells were characterized by increased transcription of several components of the IGF system (IGF-II, IGF-BP2, and IGF-BP5) and by the up-regulation of IGF-II and IGF-IR proteins. Nanog knockdown reduced IGF-IR levels, while, in turn, IGF-IR blockade inhibited Nanog expression, suggesting a positive feed-back loop between Nanog function and IGF-IR signaling (24).

### Cross-talk with p53, Sp1, and HMGA1 proteins

The IGF-IR promoter region contains multiple GC boxes, potential binding sites for Sp1, a transcription factor involved in IGF-IR promoter constitutive activity. p53, as well as Wilms’ tumor-suppressor 1, Breast Cancer 1, and other anti-oncogenes negatively regulate IGF-IR transcription, partially through functional interaction with Sp1 (88). p53 controls IGF-IR expression also at post-transcriptional level through Mdm-2. When p53 is inhibited and Mdm-2 overexpressed, Mdm-2 is redistributed from p53 to IGF-IR and this mechanism results in IGF-IR ubiquitination and degradation (89). Although less studied, IR promoter is also repressed by wild type p53 (90). These findings help explaining why the reduced activity of these anti-oncogenes may be associated with IR and IGF-IR up-regulation. Additionally, the functional link between p53 and high motility group A1 (HMGA1) proteins may play an important role in IR and IGF-IR up-regulation in CSCs.

High motility group A1 proteins are chromatin architectural factors acting as both positive and negative regulators of gene transcription. In normal cells, HMGA1 expression is restricted to embryogenesis while in transformed cells it is present at high levels and correlates with an aggressive behavior (91). Similarly to p53, also HMGA1 proteins cooperate with other signaling pathways to sustain stemness features, EMT process, and metastatic properties. Indeed, in breast and colon cancer HMGA-depleted cells, β-catenin was relocated from the nucleus to cell–cell contacts and mesenchymal marker vimentin and the Notch pathway, were downregulated. Concomitantly, HMGA1 depletion was associated with reduction in sphere self-renewal, dimension, and growth. Yet, HMGA1 was also seen to be associated with a gene signature containing genes important for EMT, stemness, metastasis, pluripotency, and strictly linked to the Wnt/β-catenin, Notch and Pin1/mutant p53 signaling pathways (92, 93).

**Figure 2:**
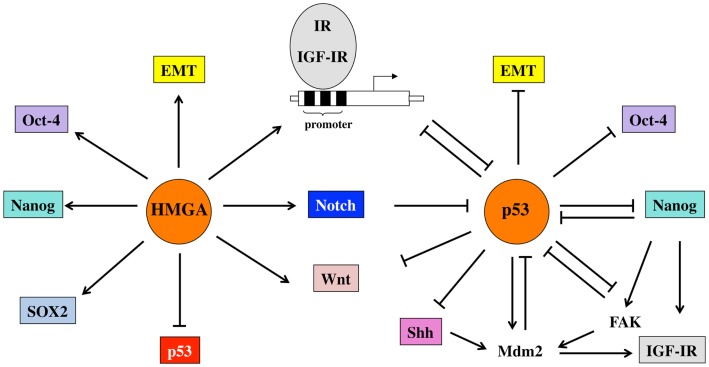
**Schematic representation of the relevant functional interactions occurring between p53 and HMGA1 proteins, the IGF system and other factors involved in pluripotency and EMT**. p53 represses the promoter activity of IR and IGF-IR and regulates both receptors also at post-transcriptional levels through the induction of Mdm-2. Mdm-2, in turn, is redistributed from p53 to IGF-IR and induces its degradation. In addition, p53 down-regulates signals important for pluripotency (Oct-4, Nanog) and stemness maintenance (Wnt, Notch, Shh). Some of these signals, in turn, modulate p53 functions. For example, Nanog inhibits p53 tumor-suppressor activity through FAK up-regulation. HMGA1 proteins, by impairing the regulatory activity of p53, but also by direct binding to promoter sequences and by stabilizing transcriptional complexes, upregulate IR and IGF-IR as well as transcriptional factors and signaling pathways linked to stemness.

Some of HMGA1 effects may be mediated by the IR/IGF-IR system. In fact, HMGA1 is able to relieve the inhibitory control of p53 on IR and IGF-IR gene transcription by interacting with the oligomerization domain of p53 and inhibiting p53 ability to oligomerize into functionally active tetramers (94). Additional mechanisms, by which HMGA1 may activate IR and IGF-IR transcription include direct interaction with the IR and IGF-IR promoter, and stabilization of transcriptional multiprotein complexes and protein–DNA interactions, including enhancement of Sp1 effect on the IR and IGF-IR promoters (95–97). A schematic representation of some of these interactions is given in Figure 2.

Therefore, tumors with inactivating mutations of p53 and/or overexpression of HMGA1 are often poorly differentiated and characterized by increased IR and IGF-IR up-regulation (91, 98).

### Cross-talk with Wnt/β-catenin signaling

The canonical Wnt signaling is initiated when a Wnt ligand engages co-receptors of the Frizzled (Fzd) and low-density lipoprotein receptor related protein (LRP) families. In the absence of Wnt, β-catenin is part of a multiprotein complex including axin, the adenomatous polyposis coli protein (APC) and GSK3β. As previously mentioned, GSK3β is a constitutively active kinase, which phosphorylates specific Ser and Thr residues of β-catenin, prompting β-catenin proteasomal degradation. Wnt binding to its co-receptors induces phosphorylation and inactivation of GSK3β, with consequent β-catenin stabilization, and translocation into the nucleus where it binds TCF/LEF (T cell factor/lymphoid-enhancer binding factor) transcription factor, a key activator of the expression of target genes controlling cell fate and self-renewal of stem/progenitors during development and specification in a variety of tissues (99–101). In particular, this pathway activated the expression of Oct-4 and Nanog, crucial regulators of cell pluripotency (102).

The tightly regulated activation of Wnt/β-catenin signaling, as well as β-catenin expression and localization may be subverted in a variety of human cancers, thus inducing an imbalance between long-term renewal and differentiation program (12, 101), which favors CSCs expansion (100). For example, in hematopoietic stem cells, Wnt activity is required for self-renewal, for the transformation of progenitor cells by certain oncogenes, and for acquiring drug-resistance (103, 104). Furthermore, the suppression of β-catenin completely abolished the development of mixed lineage leukemia stem cells, reversed leukemia stem cells to a pre-leukemia stem-like stage, and induced a greater responsiveness to GSK3β inhibitors *in vivo* and *in vitro* (105). Wnt/β-catenin signaling has also emerged as an essential pathway for self-renewal of intestinal stem cells and for their malignant transformation. Indeed, the apc gene, encoding the APC protein, is silenced in over 80% of human colon cancer (106). Loss of APC function and activating mutations of β-catenin are early steps in the pathogenesis of colorectal cancer (107). Colon CSCs are functionally characterized by high Wnt signaling activity, while the differentiated progeny of these cells own markedly lower levels of Wnt activation (108). This gradient in Wnt signaling activity is partially dependent on the microenvironment, the so-called CSC niche. Environmental factors, like hepatocyte growth factor (HGF) produced by stromal myofibroblasts, activate β-catenin-dependent transcription and subsequently CSC clonogenicity. More significantly, these stromal factors also induce the CSC phenotype in more differentiated tumor cells (109). In mammary gland, Wnt is necessary for the regulation of self-renewal and differentiation of mammary stem cells (MSCs) during embryogenesis and pregnancy in adult life. Although mutations in components of the Wnt pathway have not been identified in human breast cancers, in over 50% of them, this pathway is constitutively stabilized (110). Aberrant activation of Wnt/β-catenin signaling induces tumors of undifferentiated basal cells, while β-catenin inhibition in the mouse mammary gland blocks organ development, pregnancy-induced proliferation, and reduces the number of alveolar progenitor cells (111). Yet, Wnt/β-catenin activation has also been linked to radio- and chemo-resistance of mammary tumor-initiating cells (112).

**Figure 3:**
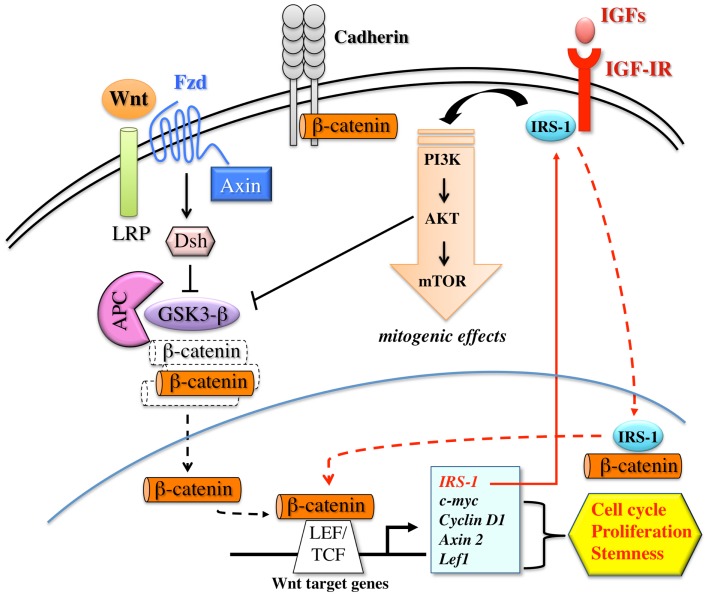
**Schematic representation of the interplay between the canonical Wnt/β-catenin pathway and IGF signaling**. The activation of the canonical Wnt/β-catenin pathway is triggered by the binding of Wnt ligands to FZD (frizzled) and LRP (LDL-related receptor protein) receptors on target cells. Receptor occupancy inhibits the kinase activity of the destruction complex, consisting of APC (adenomatous polyposis coli), Axin, Dsh (Disheveled), and GSK3β. As a consequence, β-catenin is uncoupled from the degradation complex and accumulates and translocates into the nucleus, where it binds LEF/TCF transcription factors. These events trigger efficient transcription of genes that are important regulators of cell proliferation, cell cycle progression, and stem cell fate. Wnt target genes include IRS-1, which, in turn, amplifies the IGF-IR/PI3K pathway and its mitogenic signals. IRS-1 contributes to β-catenin stabilization by activating PI3K pathway and inducing GSK3β inhibition. Furthermore, after IGF-I stimulation, IRS-1 partially translocates into the nucleus where it binds to and co-localizes with β-catenin, further contributing to TCF dependent transcription. β-catenin also exists in a cadherin-bound form and regulates cell–cell adhesion.

Several lines of evidence demonstrate that a strict cooperation between the Wnt/β-catenin and IGF signaling may contribute to carcinogenesis and cancer progression (see Figure 3). In human colon cancer cells, IGF-I stimulates tyrosine phosphorylation of β-catenin and of IRS-1 and E-cadherin, two β-catenin interacting proteins. This results in the disruption of β-catenin – E-cadherin interaction, and increased cell motility. Chiefly, IGF-I stimulates β-catenin relocation and stability through the inactivation of GSK3β (113), thus contributing to colon cell proliferation (114). Through the same pathway, IR and IGF-IR may also stimulate Oct-4 and Nanog expression. Indeed, IGF-IR was required for Oct-4 to form a complex with β-catenin and SOX2, which then activates the Nanog promoter (26).

Because IGF-IR is overexpressed in the proliferating cells at the base of the colonic crypts, which could be considered the colon tumor-initiating cells (115), and Wnt/β-catenin signaling plays a critical role in stimulating colonocyte proliferation (116), it is possible that the interplay between these two pathways could contribute to the expansion of colon tumor-initiating cells pool. This hypothesis, although requiring further validation, could be applied to other cancer models where both cascades are deregulated. This could be also the case of hepatocellular carcinoma, where insulin and IGF-I cooperate with Wnt signaling in the carcinogenesis process by stimulating TCF/LEF dependent transcription through the PI3K/GSK3β/β-catenin pathway (117). Evidence in oligodendroglial cultures cells supports the notion that β-catenin may mediate IGF-I actions on cyclin D1 expression, cell proliferation, and survival (118). All these mechanisms have also been demonstrated in cultured precursor/stem cells, where they may contribute to trigger the early steps of the carcinogenesis process (118, 119). Likewise IGF-I, IGF-II is also able to induce β-catenin relocation to the nucleus and the transcription of β-catenin/TCF-3 target genes. In parallel with these molecular events, IGF-II triggers intracellular sequestration and degradation of E-cadherin inducing rapid EMT (120).

The effect of IGF-I on β-catenin localization and stabilization is also affected by IRS-1. After IGF-I stimulation, IRS-1 is partially translocated to the nucleus, where it binds and co-localizes with β-catenin (121). On the other hand, IRS-1 itself is a downstream target gene of β-catenin, which regulates IRS-1 expression *in vivo*. The interplay between IRS-1 and β-catenin may represent another mechanism promoting cancer initiation (122), and regulating self-renewal and differentiation processes (123). Taken together, these data highlight the existence of a close connection and a positive feed-back loop between the Wnt/β-catenin and IGF signaling, which contribute to oncogenesis and EMT process.

### Cross-talk with notch signaling

The Notch system consists of four transmembrane isoform receptors (Notch-1, Notch-2, Notch-3, and Notch-4) and five canonical transmembrane ligands [Delta-like (DLL)-1, DLL-2, DLL-3, DLL-4, Jagged-1, and Jagged-2]. Notch receptor activation requires cell-to-cell interaction and starts after binding to a ligand presented by a neighboring cell. This event triggers three consecutive proteolytic cleavages, including a final cleavage by a γ-secretase complex, which produces an active and stable Notch fragment (Notch intracellular or N^IC^). N^IC^ then translocates to the nucleus and binds to the DNA-binding protein CSL (CBF-1-Suppressor of Hairless/Lag1), a constitutive transcriptional repressor, which displaces other co-repressors and recruits additional co-activators to induce the transcription of genes involved in differentiation, survival, self-renewal, and cell fate.

It is now well known that Notch pathway is aberrantly activated in a variety of tumors (124, 125). Accumulating evidence supports the involvement of the Notch pathway in CSC biology, tumor metastasis, angiogenesis, antitumor treatment resistance and EMT process. In particular, Notch signaling induces morphological changes shared by EMT and stem-like signatures, involving down-regulation of endothelial and epithelial markers (VE-cadherin, Tie1, Tie2, endothelial NO synthase, etc.), up-regulation of mesenchymal markers (A-SMA, fibronectin, N-cadherin, Vimentin, PDGF, ZEB1, Twist, Snail, Slug), and increased expression of stem-like markers [Oct-4, SOX2, Nanog, Lin28B; Ref. (126–128)]. Conversely, reduction of Notch signaling is associated with reduced proliferation, increased apoptotic susceptibility, reduced tumor-sphere formation, prevention of *in vivo* tumor implantation, and changes in the expression of stemness transcription factors [Oct-4, SOX2, Nanog; Ref. (129–132)]. Moreover, several studies have shown that only the cell subpopulation characterized by Notch activity/overexpression has the ability to form spheres *in vitro*, to self-renew, and to resist to chemotherapy (131, 133, 134).

For the aforementioned reasons, Notch has become an attractive therapeutic target to reverse EMT-dependent maintenance of CSCs, chemo-resistance, and tumor spread in several malignancies characterized by Notch pathway deregulation (110, 125).

The interaction between the Notch and the IGF-IR pathway has been firstly demonstrated by Eliasz et al. in lung adenocarcinoma cells (135). In these cells, under hypoxic conditions, Notch-1 stimulates IGF-IR transcription by regulating its promoter. Notch-1 seems to act directly, because Notch-1 downstream targets failed to modify IGF-IR expression (135). This regulatory mechanism of IGF-IR by Notch-1 is evolutionary conserved. In *Drosophila*, Notch-1 and insulin/IGF signals are required to control the number of the cap cells of the niche and to promote germ-line stem cell division (136). In *Caenorhabiditis elegans*, both pathways are intrinsically essential for surviving under unfavorable conditions such as hypoxia (137). The Notch-mediated IGF-IR regulation in hypoxic environment is important also in cancer stem biology. Indeed, hypoxia, through HIF-1α, usually promotes stem-like features providing a niche for tumor-initiating cells. These cells in hypoxic tumor areas are often dependent for growth and survival by Notch; they are also highly resistant to apoptosis and to angiogenesis inhibitors. Thus, it is possible that these tumor-initiating cells require the activation of HIF-1α/Notch/IGF-IR/Akt pathway, and may become sensitive to the antitumor effect of Notch inhibitors in combination with IGF-IR/Akt inhibitors.

The link between Notch and IGF-IR signaling has recently been confirmed in human T lymphoblastic leukemia (T-ALL) initiating cells (32), where IGF-IR was recognized as a Notch-1 target, as Notch directly up-regulated IGF-IR protein and mRNA expression in all cell lines analyzed. Notch inhibition resulted in only two- to three-folds decrease in IGF-IR expression, suggesting that Notch is not the only factor affecting IGF-IR up-regulation and signaling. However, Notch induced IGF-IR overexpression was sufficient to robustly enhance the sensitivity of T-ALL cells to IGF-I/II.

**Figure 4:**
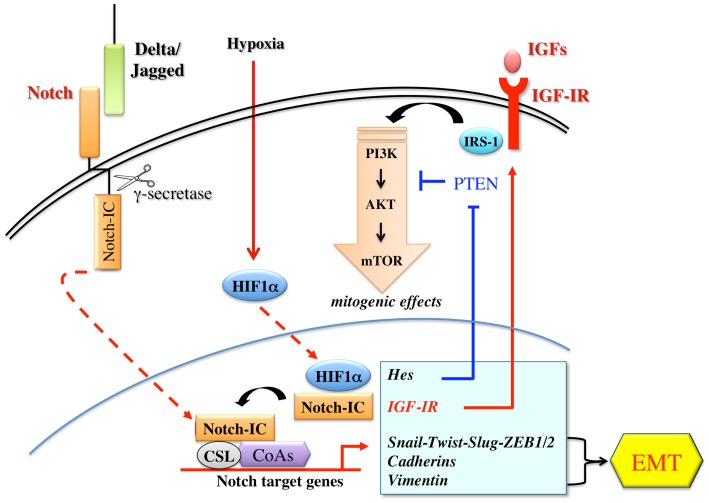
**Schematic representation of the cross-talks between the Notch and the IGF signaling**. The Notch receptor is activated by the binding to ligands (Delta-like 1/3/4; Jagged-1/2) presented by a neighboring cell. Notch activation requires three consecutive proteolytic cleavages. The final cleavage is mediated by a γ-secretase complex, which releases an active fragment (Notch-IC). Notch-IC, then, translocates into the nucleus where it interacts with the DNA-binding protein CSL (suppressor of Hairless), a constitutive transcription repressor. Upon Notch-IC binding, CSL undergoes allosteric modifications, which displace co-repressors and recruit co-activators (CoAs) to activate transcription. Notch dependent signaling induces several genes associated with differentiation, survival, stemness, and EMT. Notch target genes include Hes and Hey family of basic helix-loop-helix transcription factors, D-cyclins, transcription and growth factors relevant to EMT and to IGF-IR/PI3K pathway. Notch signaling is often and aberrantly activated by hypoxia through HIF-1-α. Under hypoxic conditions, Notch-1 stimulates IGF-IR transcription by regulating its promoter and amplifying the mitogenic effects mediated by IGF-IR/PI3K signaling.

This cross-talk with Notch may represent a general mechanism through which IGF-IR signaling influences the growth, the maintenance, and the activity of tumor-initiating cells with self-renewal capacity. A schematic representation between Notch and IGF-IR pathways is shown in Figure 4.

### Cross-talk with Shh signaling

The Shh pathway has been extensively studied for its critical role in regulating proliferation, cell fate, patterning, developmental, and cancer biology (138). During embryonic development, Shh controls pattern formation and modulates the proliferation and differentiation of progenitor/stem cells. In the adult, Shh regulates cell homeostasis through the selective activation of transcription factors involved in the maintenance of stem cells, tissue repair, and regeneration (139). Shh signaling is activated by Shh glycoprotein which binds the transmembrane receptor Patched (PTCH) to release the seven-transmembrane smoothened (SMO) protein from PTCH repression (138). SMO, in turn, activates STK36 serine/threonine kinase and stabilizes GLI family transcription factors (GLI1, GLI2, GLI3), which activate the transcription of GLI targets genes, including cyclin D1, Bcl-2, osteopontin, PTCH1, FOXL1, and JAG2. Several evidences have shown that the Shh pathway is often deregulated in various types of solid and non-solid tumors. Mutations in key components of Shh pathway, such as Patch1 and SMO, have been found in tumors from different tissues/organs, including brain, skin, pancreas, colon, lung, and prostate (140–143). Moreover, activation of Shh signaling plays a role in various steps of oncogenesis, from cancer initiation to progression and metastasis. During cancer initiation, Shh/GLI dependent gene transcription regulates stemness and self-renewal, as revealed by the up-regulation of specific CSC markers such as BMI1, CD133, and CD44 (139). During tumor growth, to fuel cancer cells, Shh pathway regulates key genes involved in cell proliferation, cell cycle progression, apoptosis, and survival (i.e., Bcl-2, TRAIL, cyclins, and c-myc) (139). Shh also contributes to cancer progression and metastasis by favoring the EMT transition through the up-regulation of Snail and the down-regulation of E-cadherin (144), thus contributing to re-program cancer cells toward a stem-like phenotype. Particular attention has been recently focused on the role of Shh pathway in the control of brain tumor stem-like cells (BTSCs), and in the genesis of brain tumors. It has been observed that GLI1 is expressed in the germinative zones of the brain, where Shh maintains a proliferative state (145), and in a variety of brain tumors including medulloblastomas of the cerebellum and gliomas of the cerebral cortex (146). In cerebellar granule precursors, which are believed to be the cells of origin for medulloblastoma, Shh signaling constitutive activation results in significantly ectopic expression of SOX2. Therefore, through SOX2 induction, Shh drives medulloblastomas cellular growth and proliferation (147). Similarly, in neural stem cells in the central nervous system, SOX2 gene is regulated by the downstream mediator of Shh signaling, GLI2 transcription factor, to sustain neural stem cells growth, expansion, and to prevent neuronal differentiation (148). In a subset of gliomas, gene-expression profile has recently confirmed the activation of Shh signaling and its key role in controlling glioma CSC self-renewal, growth, and survival. Interestingly, interference of Shh signaling with cyclopamine, a specific SMO inhibitor, or through lentiviral-mediated silencing, decreased glioma stem cell proliferation and CSC-initiated brain tumor formation in mice further confirming the important role of this pathway in controlling the behavior of BTSCs (149).

**Figure 5:**
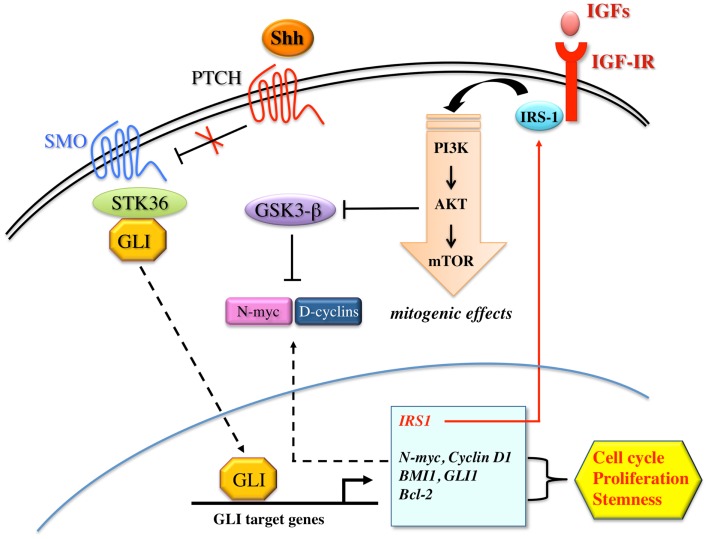
**Proposed model for the interaction between Shh and IGF signaling**. Shh binds to patched family receptors (PTCH) to releases smoothened (SMO) signal transducer from Patched-dependent suppression. Activated SMO, in turn, induces SKT36 serine/threonine kinase to stabilize GLI family members for nuclear translocation. Shh signaling activates GLI dependent transcription of target genes including N-myc, D-cyclins, BMI1, GLI1, Bcl-2, and IRS-1, all of which have been implicated in cell cycle progression, proliferation, and stemness. IRS-1, in turn, mediates IGF-IR/PI3K signaling activation, which inhibits GSK3β-mediated phosphorylation and consequent increased degradation of N-myc and D-cyclins. However, Shh may also activate IGF-IR/PI3K pathway via unknown intermediary molecules.

Using a RCAS/tv, a system, which allows a cell type specific postnatal gene transfer, Rao et al. have shown that Shh and IGF signaling synergize to induce medulloblastomas in mice (150). They found that the rate of Shh-induced tumor formation increases from 15 to 39%, when IGF-II is coexpressed, whereas no tumor formation was seen in mice injected with IGF-II alone. Furthermore, the induced tumors showed up-regulation of IRS-1 and phosphorylated IGF-IR, indicating a sustained activation of IGF signaling in these tumors. Other research groups have also reported cooperation between Shh/Ptch signaling and IGF system. In particular, it appears that IGF-II is essential for Shh-mediated medulloblastoma and rhabdomyosarcoma formation in Ptch mutant mice, and that IGF-II is a downstream transcriptional target of Shh, suggesting that IGF-II acts as a critical mediator of Shh function (151). Similarly, in cerebellar granule precursors, both IGF-I and IGF-II have synergistic proliferative effects with exogenous Shh, as Shh-induced proliferation is dependent on IGF-IR function (152). Synergism between Shh and IGF signaling has also been seen in glioma stem cells (153, 154), where Shh/GLI signaling regulates IGF dependent malignant behavior by increasing IRS-1 transcription. Indeed, genetic or pharmacological inhibition of GLI1 decreases IGF-I induced glioma stem cells self-renewal, proliferation, migration, angiogenesis, and IGF dependent MAPK activation. In addition, the blockade of Shh/GLI1 and IGF pathways sensitizes glioma stem cells to the chemotherapeutic agent temozolomide. Findings in cerebellar neural precursors are also consistent with the observation that Shh uses components of the IGF pathway to drive proliferation (155). In these cells, Shh upregulates IRS-1 by interfering with mTOR-mediated IRS-1 degradation, and by enhancing IRS-1 translation. Shh, therefore, through the newly translated and stabilized IRS-1, enhances IGF mediated survival and mitogenesis. However, this mechanism of pathway cooperation is not exclusive and there may be multiple sites and modes of interaction between Shh and IGF system. For example, IGF-IR activation enhances Shh signaling by inhibiting the GSK3β and the consequent degradation of N-myc and cyclin D1, both of which are critical mediators of the Shh pathway (156, 157). Alternatively, IGF and Shh signaling could also interact at the level of GLI1 regulation, upstream of N-myc and cyclin D1 (158), or through other components of the IGF system, such as the IGF-BPs (152). The synergistic interaction between Shh and IGF signaling is not linear and could be mediated by not yet fully known intermediary molecules. A scheme depicting the interplay between Shh and the IGF system is shown in Figure 5. Collectively, these results, although obtained in neural cells and still needing validation in other organs, provide strong evidence that Shh/GLI signaling synergizes with the IGF system.

## Conclusion and Perspectives

It is well established that IGF signaling plays a crucial role in cancer, and that both several oncogenes and anti-oncogenes regulates IGF-IR expression and signaling to sustain cancer progression (159). This notion has stimulated the development of IGF-IR blocking therapies, with the aim to block cancer growth with minimal side effects on glucose metabolism. However, IGF-IR overexpression is only part of the complex IGF signaling deregulation occurring in cancer, and it has soon become evident that overactivation of other components of the IGF system, including the IGF-II/IR-A circuitry and systemic hyperinsulinemia, may induce resistance to IGF-IR blocking therapies (9, 65). The disappointing results of clinical trials with anti-IGF-IR antibodies have not disproved that the IGF signaling plays a key role in cancer but have clearly indicated that this system is a difficult one to target because of the several levels of homeostatic control.

Noteworthy, several recent studies now support the concept that IGF signaling not only plays a proliferative and antiapoptotic role in cancer cells, but also sustains cancer cell EMT and stemness, two key features of cancer cell renewal and metastatic phenotype. In fact, on one hand, the IGF signaling appears to stimulate the transcription factors of the ZEB and the Snail family implicated in the EMT program, and, on the other hand, it strictly interacts with transcription factors (e.g., Oct-4, SOX2, Nanog, p53, HMGA1 proteins) and specific signaling pathways (the Wnt/β-catenin, the Notch, and the Shh pathways) classically involved in cell stemness. These interactions occur through several components and multiple modalities, establishing an intricate network characterized by multiple positive feed-back mechanisms. These findings may open new hope for cancer treatment, by showing that targeting the IGF system is still a viable option in several malignancies, but we need to target at the same time other components of this complex signaling network especially cooperating in the EMT process and cell stemness.

## Conflict of Interest Statement

The authors declare that the research was conducted in the absence of any commercial or financial relationships that could be construed as a potential conflict of interest.

## References

[1] Brogiolo W Stocker H Ikeya T Rintelen F Fernandez R Hafen E An evolutionarily conserved function of the *Drosophila* insulin receptor and insulin-like peptides in growth control. Curr Biol (2001) 11:213–21 10.1016/S0960-9822(01)00068-9 11250149

[2] Valentinis B Baserga R IGF-I receptor signalling in transformation and differentiation. Mol Pathol (2001) 54:133–7 10.1136/mp.54.3.133 11376123 PMC1187050

[3] Belfiore A Frasca F Pandini G Sciacca L Vigneri R Insulin receptor isoforms and insulin receptor/insulin-like growth factor receptor hybrids in physiology and disease. Endocr Rev (2009) 30:586–623 10.1210/er.2008-0047 19752219

[4] Malaguarnera R Sacco A Voci C Pandini G Vigneri R Belfiore A Proinsulin binds with high affinity the insulin receptor isoform A and predominantly activates the mitogenic pathway. Endocrinology (2012) 153(5):2152–63 10.1210/en.2011-1843 22355074

[5] El-Shewy HM Luttrell LM Insulin-like growth factor-2/mannose-6 phosphate receptors. Vitam Horm (2009) 80:667–97 10.1016/S0083-6729(08)00624-9 19251055

[6] Gallagher EJ LeRoith D Mini review: IGF, insulin, and cancer. Endocrinology (2011) 152:2546–51 10.1210/en.2011-0231 21540285

[7] Baserga R Peruzzi F Reiss K The IGF-1 receptor in cancer biology. Int J Cancer (2003) 107:873–7 10.1002/ijc.11487 14601044

[8] LeRoith D Roberts CT Jr The insulin-like growth factor system and cancer. Cancer Lett (2003) 195:127–37 10.1016/S0304-3835(03)00159-9 12767520

[9] Pollak M Targeting insulin and insulin-like growth factor signalling in oncology. Curr Opin Pharmacol (2008) 8:384–92 10.1016/j.coph.2008.07.004 18674638

[10] Baserga R The decline and fall of the IGF-I receptor. J Cell Physiol (2013) 228:675–9 10.1002/jcp.24217 22926508

[11] Reya T Morrison SJ Clarke MF Weissman IL Stem cells, cancer, and cancer stem cells. Nature (2001) 414:105–11 10.1038/35102167 11689955

[12] Clevers H Wnt/beta-catenin signaling in development and disease. Cell (2006) 127:469–80 10.1016/j.cell.2006.10.018 17081971

[13] Wicha MS Liu S Dontu G Cancer stem cells: an old idea – a paradigm shift. Cancer Res (2006) 66:1883–6 10.1158/0008-5472.CAN-05-3153 16488983

[14] Mani SA Guo W Liao M-J Eaton EN Ayyanan A Zhou AY The epithelial-mesenchymal transition generates cells with properties of stem cells. Cell (2008) 133:704–15 10.1016/j.cell.2008.03.027 18485877 PMC2728032

[15] Bendall SC Stewart MH Menendez P George D Vijayaragavan K Werbowetski-Ogilvie T IGF and FGF cooperatively establish the regulatory stem cell niche of pluripotent human cells in vitro. Nature (2007) 448:1015–21 10.1038/nature06027 17625568

[16] McDevitt TC Laflamme MA Murry CE Proliferation of cardiomyocytes derived from human embryonic stem cells is mediated via the IGF/PI 3-kinase/Akt signaling pathway. J Mol Cell Cardiol (2005) 39:865–73 10.1016/j.yjmcc.2005.09.007 16242146 PMC3505759

[17] Stachelscheid H Ibrahim H Koch L Schmitz A Tscharntke M Wunderlich FT Epidermal insulin/IGF-1 signalling control interfollicular morphogenesis and proliferative potential through Rac activation. EMBO J (2008) 27:2091–101 10.1038/emboj.2008.141 18650937 PMC2516889

[18] Yan YP Sailor KA Vemuganti R Dempsey RJ Insulin-like growth factor-1 is an endogenous mediator of focal ischemia-induced neural progenitor proliferation. Eur J Neurosci (2006) 24:45–54 10.1111/j.1460-9568.2006.04872.x 16882007

[19] Savarese TM Strohsnitter WC Low HP Liu Q Baik I Okulicz W Correlation of umbilical cord blood hormones and growth factors with stem cell potential: implications for the prenatal origin of breast cancer hypothesis. Breast Cancer Res (2007) 9:R29 10.1186/bcr1674 17501995 PMC1929091

[20] Ivanova NB Dimos JT Schaniel C Hackney JA Moore KA Lemischka IR A stem cell molecular signature. Science (2002) 298:601–4 10.1126/science.1073823 12228721

[21] Ziegler AN Schneider JS Qin M Tyler WA Pintar JE Fraidenraich D IGF-II promotes stemness of neural restricted precursors. Stem Cells (2012) 30:1265–76 10.1002/stem.1095 22593020 PMC5581406

[22] Ziegler AN Chidambaram S Forbes BE Wood TL Levison SW IGF-II and IGF-II analogs with enhanced insulin receptor-A binding affinity promote neural stem cell expansion. J Biol Chem (2014). Available from: http://www.ncbi.nlm.nih.gov/pubmed/24398690 10.1074/jbc.M113.537597 PMC3931023 24398690

[23] Malaguarnera R Frasca F Garozzo A Giani F Pandini G Vella V Insulin receptor isoforms and insulin-like growth factor receptor in human follicular cell precursors from papillary thyroid cancer and normal thyroid. J Clin Endocrinol Metab (2011) 96:766–74 10.1210/jc.2010-1255 21123448

[24] Shan J Shen J Liu L Xia F Xu C Duan G Nanog regulates self-renewal of cancer stem cells through the insulin-like growth factor pathway in human hepatocellular carcinoma. Hepatology (2012) 56:1004–14 10.1002/hep.25745 22473773

[25] Chang W-W Lin R-J Yu J Chang W-Y Fu C-H Lai AC-Y The expression and significance of insulin-like growth factor-1 receptor and its pathway on breast cancer stem/progenitors. Breast Cancer Res (2013) 15:R39 10.1186/bcr3423 23663564 PMC3706809

[26] Xu C Xie D Yu S-C Yang X-J He L-R Yang J β-Catenin/POU5F1/SOX2 transcription factor complex mediates IGF-I receptor signaling and predicts poor prognosis in lung adenocarcinoma. Cancer Res (2013) 73:3181–9 10.1158/0008-5472.CAN-12-4403 23539445

[27] Dallas NA Xia L Fan F Gray MJ Gaur P van Buren G II Chemoresistant colorectal cancer cells, the cancer stem cell phenotype, and increased sensitivity to insulin-like growth factor-I receptor inhibition. Cancer Res (2009) 69:1951–7 10.1158/0008-5472.CAN-08-2023 19244128 PMC3198868

[28] Hart LS Dolloff NG Dicker DT Koumenis C Christensen JG Grimberg A Human colon cancer stem cells are enriched by insulin-like growth factor-1 and are sensitive to figitumumab. Cell Cycle (2011) 10:2331–8 10.4161/cc.10.14.16418 21720213 PMC3322474

[29] Bodzin AS Wei Z Hurtt R Gu T Doria C Gefitinib resistance in HCC Mahlavu cells: upregulation of CD133 expression, activation of IGF-1R signaling pathway, and enhancement of IGF-1R nuclear translocation. J Cell Physiol (2012) 227:2947–52 10.1002/jcp.23041 21959795

[30] Aleksic T Chitnis MM Perestenko OV Gao S Thomas PH Turner GD Type 1 insulin-like growth factor receptor translocates to the nucleus of human tumor cells. Cancer Res (2010) 70:6412–9 10.1158/0008-5472.CAN-10-0052 20710042 PMC2981028

[31] Sarfstein R Pasmanik-Chor M Yeheskel A Edry L Shomron N Warman N Insulin-like growth factor-I receptor (IGF-IR) translocates to nucleus and autoregulates IGF-IR gene expression in breast cancer cells. J Biol Chem (2012) 287:2766–76 10.1074/jbc.M111.281782 22128190 PMC3268434

[32] Medyouf H Gusscott S Wang H Tseng J-C Wai C Nemirovsky O High-level IGF1R expression is required for leukemia-initiating cell activity in T-ALL and is supported by notch signaling. J Exp Med (2011) 208:1809–22 10.1084/jem.20110121 21807868 PMC3171095

[33] Jenkins CR Shevchuk OO Giambra V Lam SH Carboni JM Gottardis MM IGF signaling contributes to malignant transformation of hematopoietic progenitors by the MLL-AF9 oncoprotein. Exp Hematol (2012) 40:715.e–23.e 10.1016/j.exphem.2012.05.003 22613471

[34] Fang X Cai Y Liu J Wang Z Wu Q Zhang Z Twist2 contributes to breast cancer progression by promoting an epithelial-mesenchymal transition and cancer stem-like cell self-renewal. Oncogene (2011) 30:4707–20 10.1038/onc.2011.181 21602879

[35] Kurrey NK Jalgaonkar SP Joglekar AV Ghanate AD Chaskar PD Doiphode RY Snail and slug mediate radioresistance and chemoresistance by antagonizing p53-mediated apoptosis and acquiring a stem-like phenotype in ovarian cancer cells. Stem Cells (2009) 27:2059–68 10.1002/stem.154 19544473

[36] Hwang W-L Yang M-H Tsai M-L Lan H-Y Su S-H Chang S-C SNAIL regulates interleukin-8 expression, stem cell-like activity, and tumorigenicity of human colorectal carcinoma cells. Gastroenterology (2011) 141:.e1–5 10.1053/j.gastro.2011.04.008 21640118

[37] Peinado H Olmeda D Cano A Snail, Zeb and bHLH factors in tumour progression: an alliance against the epithelial phenotype? Nat Rev Cancer (2007) 7:415–28 10.1038/nrc2131 17508028

[38] Wellner U Schubert J Burk UC Schmalhofer O Zhu F Sonntag A The EMT-activator ZEB1 promotes tumorigenicity by repressing stemness-inhibiting microRNAs. Nat Cell Biol (2009) 11:1487–95 10.1038/ncb1998 19935649

[39] Dupont J Fernandez AM Glackin CA Helman L LeRoith D Insulin-like growth factor 1 (IGF-1)-induced twist expression is involved in the anti-apoptotic effects of the IGF-1 receptor. J Biol Chem (2001) 276:26699–707 10.1074/jbc.M102664200 11323435

[40] Graham TR Zhau HE Odero-Marah VA Osunkoya AO Kimbro KS Tighiouart M Insulin-like growth factor-I-dependent up-regulation of ZEB1 drives epithelial-to-mesenchymal transition in human prostate cancer cells. Cancer Res (2008) 68:2479–88 10.1158/0008-5472.CAN-07-2559 18381457

[41] Yost C Torres M Miller JR Huang E Kimelman D Moon RT The axis-inducing activity, stability, and subcellular distribution of beta-catenin is regulated in *Xenopus* embryos by glycogen synthase kinase 3. Genes Dev (1996) 10:1443–54 10.1101/gad.10.12.1443 8666229

[42] Diehl JA Cheng M Roussel MF Sherr CJ Glycogen synthase kinase-3beta regulates cyclin D1 proteolysis and subcellular localization. Genes Dev (1998) 12:3499–511 10.1101/gad.12.22.3499 9832503 PMC317244

[43] Gupta PB Kuperwasser C Brunet J-P Ramaswamy S Kuo W-L Gray JW The melanocyte differentiation program predisposes to metastasis after neoplastic transformation. Nat Genet (2005) 37:1047–54 10.1038/ng1634 16142232 PMC1694635

[44] Zhou BP Deng J Xia W Xu J Li YM Gunduz M Dual regulation of snail by GSK-3beta-mediated phosphorylation in control of epithelial-mesenchymal transition. Nat Cell Biol (2004) 6:931–40 10.1038/ncb1173 15448698

[45] Bachelder RE Yoon S-O Franci C de Herreros AG Mercurio AM Glycogen synthase kinase-3 is an endogenous inhibitor of snail transcription: implications for the epithelial-mesenchymal transition. J Cell Biol (2005) 168:29–33 10.1083/jcb.200409067 15631989 PMC2171685

[46] Kao S-H Wang W-L Chen C-Y Chang Y-L Wu Y-Y Wang Y-T GSK3β controls epithelial-mesenchymal transition and tumor metastasis by CHIP-mediated degradation of Slug. Oncogene (2013). Available from: http://www.ncbi.nlm.nih.gov/pubmed/23851495 10.1038/onc.2013.279 PMC4096338 23851495

[47] John JK Paraiso KHT Rebecca VW Cantini LP Abel EV Pagano N GSK3β inhibition blocks melanoma cell/host interactions by downregulating N-cadherin expression and decreasing FAK phosphorylation. J Invest Dermatol (2012) 132:2818–27 10.1038/jid.2012.237 22810307 PMC3479306

[48] Papkoff J Aikawa M WNT-1 and HGF regulate GSK3 beta activity and beta-catenin signaling in mammary epithelial cells. Biochem Biophys Res Commun (1998) 247:851–8 10.1006/bbrc.1998.8888 9647782

[49] Davies MA Stemke-Hale K Tellez C Calderone TL Deng W Prieto VG A novel AKT3 mutation in melanoma tumours and cell lines. Br J Cancer (2008) 99:1265–8 10.1038/sj.bjc.6604637 18813315 PMC2570525

[50] Paraiso KHT Xiang Y Rebecca VW Abel EV Chen YA Munko AC PTEN loss confers BRAF inhibitor resistance to melanoma cells through the suppression of BIM expression. Cancer Res (2011) 71:2750–60 10.1158/0008-5472.CAN-10-2954 21317224 PMC3070772

[51] Guvakova MA Surmacz E The activated insulin-like growth factor I receptor induces depolarization in breast epithelial cells characterized by actin filament disassembly and tyrosine dephosphorylation of FAK, Cas, and paxillin. Exp Cell Res (1999) 251:244–55 10.1006/excr.1999.4566 10438590

[52] Julien S Puig I Caretti E Bonaventure J Nelles L van Roy F Activation of NF-kappaB by Akt upregulates snail expression and induces epithelium mesenchyme transition. Oncogene (2007) 26:7445–56 10.1038/sj.onc.1210546 17563753

[53] Chua HL Bhat-Nakshatri P Clare SE Morimiya A Badve S Nakshatri H NF-kappaB represses E-cadherin expression and enhances epithelial to mesenchymal transition of mammary epithelial cells: potential involvement of ZEB-1 and ZEB-2. Oncogene (2007) 26:711–24 10.1038/sj.onc.1209808 16862183

[54] Barberà MJ Puig I Domínguez D Julien-Grille S Guaita-Esteruelas S Peiró S Regulation of snail transcription during epithelial to mesenchymal transition of tumor cells. Oncogene (2004) 23:7345–54 10.1038/sj.onc.1207990 15286702

[55] Wu Y Deng J Rychahou PG Qiu S Evers BM Zhou BP Stabilization of snail by NF-kappaB is required for inflammation-induced cell migration and invasion. Cancer Cell (2009) 15:416–28 10.1016/j.ccr.2009.03.016 19411070 PMC2881229

[56] Irie HY Pearline RV Grueneberg D Hsia M Ravichandran P Kothari N Distinct roles of Akt1 and Akt2 in regulating cell migration and epithelial-mesenchymal transition. J Cell Biol (2005) 171:1023–34 10.1083/jcb.200505087 16365168 PMC2171329

[57] Dearth RK Cui X Kim H-J Kuiatse I Lawrence NA Zhang X Mammary tumorigenesis and metastasis caused by overexpression of insulin receptor substrate 1 (IRS-1) or IRS-2. Mol Cell Biol (2006) 26:9302–14 10.1128/MCB.00260-06 17030631 PMC1698542

[58] Sorokin AV Chen J MEMO1, a new IRS1-interacting protein, induces epithelial-mesenchymal transition in mammary epithelial cells. Oncogene (2013) 32:3130–8 10.1038/onc.2012.327 22824790

[59] Sivakumar R Koga H Selvendiran K Maeyama M Ueno T Sata M Autocrine loop for IGF-I receptor signaling in SLUG-mediated epithelial-mesenchymal transition. Int J Oncol (2009) 34:329–38 10.3892/ijo_00000155 19148466

[60] Frolov MV Dyson NJ Molecular mechanisms of E2F-dependent activation and pRB-mediated repression. J Cell Sci (2004) 117:2173–81 10.1242/jcs.01227 15126619

[61] Sacco A Morcavallo A Pandini G Vigneri R Belfiore A Differential signaling activation by insulin and insulin-like growth factors I and II upon binding to insulin receptor isoform A. Endocrinology (2009) 150:3594–602 10.1210/en.2009-0377 19443570

[62] Zhao H Desai V Wang J Epstein DM Miglarese M Buck E Epithelial-mesenchymal transition predicts sensitivity to the dual IGF-1R/IR inhibitor OSI-906 in hepatocellular carcinoma cell lines. Mol Cancer Ther (2012) 11:503–13 10.1158/1535-7163.MCT-11-0327 22161861

[63] Vella V Pandini G Sciacca L Mineo R Vigneri R Pezzino V A novel autocrine loop involving IGF-II and the insulin receptor isoform-A stimulates growth of thyroid cancer. J Clin Endocrinol Metab (2002) 87:245–54 10.1210/jc.87.1.245 11788654

[64] Yang Y Yee D Targeting insulin and insulin-like growth factor signaling in breast cancer. J Mammary Gland Biol Neoplasia (2012) 17:251–61 10.1007/s10911-012-9268-y 23054135 PMC3534944

[65] Garofalo C Manara MC Nicoletti G Marino MT Lollini PL Astolfi A Efficacy of and resistance to anti-IGF-1R therapies in Ewing’s sarcoma is dependent on insulin receptor signaling. Oncogene (2011) 30(24):2730–40 10.1038/onc.2010.640 21278796

[66] Pandini G Frasca F Mineo R Sciacca L Vigneri R Belfiore A Insulin/insulin-like growth factor I hybrid receptors have different biological characteristics depending on the insulin receptor isoform involved. J Biol Chem (2002) 277:39684–95 10.1074/jbc.M202766200 12138094

[67] Andres SF Simmons JG Mah AT Santoro MA Van Landeghem L Lund PK Insulin receptor isoform switching in intestinal stem cells, progenitors, differentiated lineages and tumors: evidence that IR-B limits proliferation. J Cell Sci (2013) 126:5645–56 10.1242/jcs.132985 24127567 PMC3860310

[68] Sciacca L Prisco M Wu A Belfiore A Vigneri R Baserga R Signaling differences from the A and B isoforms of the insulin receptor (IR) in 32D cells in the presence or absence of IR substrate-1. Endocrinology (2003) 144:2650–8 10.1210/en.2002-0136 12746329

[69] Liu J Visser-Grieve S Boudreau J Yeung B Lo S Chamberlain G Insulin activates the insulin receptor to downregulate the PTEN tumour suppressor. Oncogene (2013). Available from: http://www.ncbi.nlm.nih.gov/pubmed/23995781 10.1038/onc.2013.347 23995781

[70] Kosaki A Webster NJ Effect of dexamethasone on the alternative splicing of the insulin receptor mRNA and insulin action in HepG2 hepatoma cells. J Biol Chem (1993) 268:21990–6 8408055

[71] Warzecha CC Carstens RP Complex changes in alternative pre-mRNA splicing play a central role in the epithelial-to-mesenchymal transition (EMT). Semin Cancer Biol (2012) 22:417–27 10.1016/j.semcancer.2012.04.003 22548723 PMC3413750

[72] Talukdar I Sen S Urbano R Thompson J Yates JR Webster NJG hnRNP A1 and hnRNP F modulate the alternative splicing of exon 11 of the insulin receptor gene. PLoS One (2011) 6:e27869 10.1371/journal.pone.0027869 22132154 PMC3223206

[73] Chettouh H Fartoux L Aoudjehane L Wendum D Clapéron A Chrétien Y Mitogenic insulin receptor-A is overexpressed in human hepatocellular carcinoma due to EGFR-mediated dysregulation of RNA splicing factors. Cancer Res (2013) 73:3974–86 10.1158/0008-5472.CAN-12-3824 23633480

[74] Takahashi K Tanabe K Ohnuki M Narita M Ichisaka T Tomoda K Induction of pluripotent stem cells from adult human fibroblasts by defined factors. Cell (2007) 131:861–72 10.1016/j.cell.2007.11.019 18035408

[75] Yu J Vodyanik MA Smuga-Otto K Antosiewicz-Bourget J Frane JL Tian S Induced pluripotent stem cell lines derived from human somatic cells. Science (2007) 318:1917–20 10.1126/science.1151526 18029452

[76] Hong H Takahashi K Ichisaka T Aoi T Kanagawa O Nakagawa M Suppression of induced pluripotent stem cell generation by the p53-p21 pathway. Nature (2009) 460:1132–5 10.1038/nature08235 19668191 PMC2917235

[77] Zbinden M Duquet A Lorente-Trigos A Ngwabyt S-N Borges I Ruiz i Altaba A NANOG regulates glioma stem cells and is essential in vivo acting in a cross-functional network with GLI1 and p53. EMBO J (2010) 29:2659–74 10.1038/emboj.2010.137 20581802 PMC2928692

[78] Pan G Thomson JA Nanog and transcriptional networks in embryonic stem cell pluripotency. Cell Res (2007) 17:42–9 10.1038/sj.cr.7310125 17211451

[79] Golubovskaya VM FAK and Nanog cross talk with p53 in cancer stem cells. Anticancer Agents Med Chem (2013) 13:576–80 10.2174/1871520611313040006 22934707 PMC3625462

[80] Schubert J Brabletz T p53 Spreads out further: suppression of EMT and stemness by activating miR-200c expression. Cell Res (2011) 21:705–7 10.1038/cr.2011.62 21483453 PMC3203673

[81] Kogan-Sakin I Tabach Y Buganim Y Molchadsky A Solomon H Madar S Mutant p53(R175H) upregulates Twist1 expression and promotes epithelial-mesenchymal transition in immortalized prostate cells. Cell Death Differ (2011) 18:271–81 10.1038/cdd.2010.94 20689556 PMC3131887

[82] Reya T Clevers H Wnt signalling in stem cells and cancer. Nature (2005) 434:843–50 10.1038/nature03319 15829953

[83] Fre S Vignjevic D Schoumacher M Duffy SL Janssen K-P Robine S Epithelial morphogenesis and intestinal cancer: new insights in signaling mechanisms. Adv Cancer Res (2008) 100:85–111 10.1016/S0065-230X(08)00003-1 18620093

[84] Takahashi K Suzuki K Association of insulin-like growth-factor-I-induced DNA synthesis with phosphorylation and nuclear exclusion of p53 in human breast cancer MCF-7 cells. Int J Cancer (1993) 55:453–8 10.1002/ijc.2910550322 8375929

[85] Mimeault M Batra SK Altered gene products involved in the malignant reprogramming of cancer stem/progenitor cells and multitargeted therapies. Mol Aspects Med (2013). Available from: http://www.ncbi.nlm.nih.gov/pubmed/23994756 10.1016/j.mam.2013.08.001 PMC3938987 23994756

[86] Catrina SB Botusan IR Rantanen A Catrina AI Pyakurel P Savu O Hypoxia-inducible factor-1alpha and hypoxia-inducible factor-2alpha are expressed in Kaposi sarcoma and modulated by insulin-like growth factor-I. Clin Cancer Res (2006) 12:4506–14 10.1158/1078-0432.CCR-05-2473 16899596

[87] Semenza GL HIF-1 mediates metabolic responses to intratumoral hypoxia and oncogenic mutations. J Clin Invest (2013) 123:3664–71 10.1172/JCI67230 23999440 PMC3754249

[88] Werner H Maor S The insulin-like growth factor-I receptor gene: a downstream target for oncogene and tumor suppressor action. Trends Endocrinol Metab (2006) 17:236–42 10.1016/j.tem.2006.06.007 16815029

[89] Girnita L Girnita A Larsson O Mdm2-dependent ubiquitination and degradation of the insulin-like growth factor 1 receptor. Proc Natl Acad Sci U S A (2003) 100:8247–52 10.1073/pnas.1431613100 12821780 PMC166214

[90] Webster NJ Resnik JL Reichart DB Strauss B Haas M Seely BL Repression of the insulin receptor promoter by the tumor suppressor gene product p53: a possible mechanism for receptor overexpression in breast cancer. Cancer Res (1996) 56:2781–8 8665514

[91] Shah SN Resar LMS High mobility group A1 and cancer: potential biomarker and therapeutic target. Histol Histopathol (2012) 27:567–79 22419021 10.14670/HH-27.567

[92] Pegoraro S Ros G Piazza S Sommaggio R Ciani Y Rosato A HMGA1 promotes metastatic processes in basal-like breast cancer regulating EMT and stemness. Oncotarget (2013) 4:1293–308 23945276 10.18632/oncotarget.1136 PMC3787158

[93] Belton A Gabrovsky A Bae YK Reeves R Iacobuzio-Donahue C Huso DL HMGA1 induces intestinal polyposis in transgenic mice and drives tumor progression and stem cell properties in colon cancer cells. PLoS One (2012) 7:e30034 10.1371/journal.pone.0030034 22276142 PMC3262796

[94] Frasca F Rustighi A Malaguarnera R Altamura S Vigneri P Del Sal G HMGA1 inhibits the function of p53 family members in thyroid cancer cells. Cancer Res (2006) 66:2980–9 10.1158/0008-5472.CAN-05-2637 16540646

[95] Foti D Iuliano R Chiefari E Brunetti A A nucleoprotein complex containing Sp1, C/EBP beta, and HMGI-Y controls human insulin receptor gene transcription. Mol Cell Biol (2003) 23:2720–32 10.1128/MCB.23.8.2720-2732.2003 12665574 PMC152545

[96] Aiello A Pandini G Sarfstein R Werner H Manfioletti G Vigneri R HMGA1 protein is a positive regulator of the insulin-like growth factor-I receptor gene. Eur J Cancer (2010) 46:1919–26 10.1016/j.ejca.2010.02.050 20335021

[97] Sarfstein R Belfiore A Werner H Identification of insulin-like growth factor-I receptor (IGF-IR) gene promoter-binding proteins in estrogen receptor (ER)-positive and ER-depleted breast cancer cells. Cancers (Basel) (2010) 2:233–61 10.3390/cancers2020233 24281069 PMC3835077

[98] Hudelist G Wagner T Rosner M Fink-Retter A Gschwantler-Kaulich D Czerwenka K Intratumoral IGF-I protein expression is selectively upregulated in breast cancer patients with BRCA1/2 mutations. Endocr Relat Cancer (2007) 14:1053–62 10.1677/ERC-06-0075 18045956

[99] Zechner D Fujita Y Hülsken J Müller T Walther I Taketo MM beta-Catenin signals regulate cell growth and the balance between progenitor cell expansion and differentiation in the nervous system. Dev Biol (2003) 258:406–18 10.1016/S0012-1606(03)00123-4 12798297

[100] Reya T Wnt signalling in stem cells and cancer. Nature (2005) 434:843–50 10.1038/nature03319 15829953

[101] Moon RT Kohn AD De Ferrari GV Kaykas A WNT and beta-catenin signalling: diseases and therapies. Nat Rev Genet (2004) 5:691–701 10.1038/nrg1427 15372092

[102] Sato N Meijer L Skaltsounis L Greengard P Brivanlou AH Maintenance of pluripotency in human and mouse embryonic stem cells through activation of Wnt signaling by a pharmacological GSK-3-specific inhibitor. Nat Med (2004) 10:55–63 10.1038/nm979 14702635

[103] Wang Y Krivtsov AV Sinha AU North TE Goessling W Feng Z The Wnt/beta-catenin pathway is required for the development of leukemia stem cells in AML. Science (2010) 327:1650–3 10.1126/science.1186624 20339075 PMC3084586

[104] Scheller M Huelsken J Rosenbauer F Taketo MM Birchmeier W Tenen DG Hematopoietic stem cell and multilineage defects generated by constitutive beta-catenin activation. Nat Immunol (2006) 7:1037–47 10.1038/ni1387 16951686

[105] Yeung J Esposito MT Gandillet A Zeisig BB Griessinger E Bonnet D β-Catenin mediates the establishment and drug resistance of MLL leukemic stem cells. Cancer Cell (2010) 18:606–18 10.1016/j.ccr.2010.10.032 21156284

[106] Polakis P Wnt signaling and cancer. Genes Dev (2000) 14:1837–51 10.1101/gad.14.15.1837 10921899

[107] Barker N Ridgway RA van Es JH van de Wetering M Begthel H van den Born M Crypt stem cells as the cells-of-origin of intestinal cancer. Nature (2009) 457:608–11 10.1038/nature07602 19092804

[108] de Sousa EMF Vermeulen L Richel D Medema JP Targeting Wnt signaling in colon cancer stem cells. Clin Cancer Res (2011) 17:647–53 10.1158/1078-0432.CCR-10-1204 21159886

[109] Vermeulen L De Sousa E Melo F van der Heijden M Cameron K de Jong JH Wnt activity defines colon cancer stem cells and is regulated by the microenvironment. Nat Cell Biol (2010) 12:468–76 10.1038/ncb2048 20418870

[110] Harrison H Farnie G Brennan KR Clarke RB Breast cancer stem cells: something out of notching? Cancer Res (2010) 70:8973–6 10.1158/0008-5472.CAN-10-1559 21045140

[111] Grigoryan T Wend P Klaus A Birchmeier W Deciphering the function of canonical Wnt signals in development and disease: conditional loss- and gain-of-function mutations of beta-catenin in mice. Genes Dev (2008) 22:2308–41 10.1101/gad.1686208 18765787 PMC2749675

[112] Wang H Zhang G Zhang H Zhang F Zhou B Ning F Acquisition of epithelial-mesenchymal transition phenotype and cancer stem cell-like properties in cisplatin-resistant lung cancer cells through AKT/β-catenin/snail signaling pathway. Eur J Pharmacol (2013) 723C:156–66 10.1016/j.ejphar.2013.12.004 24333218

[113] Playford MP Bicknell D Bodmer WF Macaulay VM Insulin-like growth factor 1 regulates the location, stability, and transcriptional activity of beta-catenin. Proc Natl Acad Sci U S A (2000) 97:12103–8 10.1073/pnas.210394297 11035789 PMC17301

[114] Vanamala J Reddivari L Radhakrishnan S Tarver C Resveratrol suppresses IGF-1 induced human colon cancer cell proliferation and elevates apoptosis via suppression of IGF-1R/Wnt and activation of p53 signaling pathways. BMC Cancer (2010) 10:238 10.1186/1471-2407-10-238 20504360 PMC2891636

[115] Davies M Gupta S Goldspink G Winslet M The insulin-like growth factor system and colorectal cancer: clinical and experimental evidence. Int J Colorectal Dis (2006) 21:201–8 10.1007/s00384-005-0776-8 15959790

[116] Polakis P The many ways of Wnt in cancer. Curr Opin Genet Dev (2007) 17:45–51 10.1016/j.gde.2006.12.007 17208432

[117] Desbois-Mouthon C Cadoret A Blivet-Van Eggelpoël MJ Bertrand F Cherqui G Perret C Insulin and IGF-1 stimulate the beta-catenin pathway through two signalling cascades involving GSK-3beta inhibition and Ras activation. Oncogene (2001) 20:252–9 10.1038/sj.onc.1204064 11313952

[118] Ye P Hu Q Liu H Yan Y D’Ercole AJ beta-catenin mediates insulin-like growth factor-I actions to promote cyclin D1 mRNA expression, cell proliferation and survival in oligodendroglial cultures. Glia (2010) 58:1031–41 10.1002/glia.20984 20235220 PMC2917840

[119] Gehmert S Sadat S Song Y-H Yan Y Alt E The anti-apoptotic effect of IGF-1 on tissue resident stem cells is mediated via PI3-kinase dependent secreted frizzled related protein 2 (Sfrp2) release. Biochem Biophys Res Commun (2008) 371:752–5 10.1016/j.bbrc.2008.04.151 18466761

[120] Morali OG Delmas V Moore R Jeanney C Thiery JP Larue L IGF-II induces rapid beta-catenin relocation to the nucleus during epithelium to mesenchyme transition. Oncogene (2001) 20:4942–50 10.1038/sj.onc.1204660 11526479

[121] Chen J Wu A Sun H Drakas R Garofalo C Cascio S Functional significance of type 1 insulin-like growth factor-mediated nuclear translocation of the insulin receptor substrate-1 and beta-catenin. J Biol Chem (2005) 280:29912–20 10.1074/jbc.M504516200 15967802

[122] Bommer GT Feng Y Iura A Giordano TJ Kuick R Kadikoy H IRS1 regulation by Wnt/beta-catenin signaling and varied contribution of IRS1 to the neoplastic phenotype. J Biol Chem (2010) 285:1928–38 10.1074/jbc.M109.060319 19843521 PMC2804351

[123] Rubin R Arzumanyan A Soliera AR Ross B Peruzzi F Prisco M Insulin receptor substrate (IRS)-1 regulates murine embryonic stem (mES) cells self-renewal. J Cell Physiol (2007) 213:445–53 10.1002/jcp.21185 17620314 PMC3760688

[124] Espinoza I Miele L Deadly crosstalk: notch signaling at the intersection of EMT and cancer stem cells. Cancer Lett (2013) 341:41–5 10.1016/j.canlet.2013.08.027 23973264

[125] Leong KG Karsan A Recent insights into the role of notch signaling in tumorigenesis. Blood (2006) 107:2223–33 10.1182/blood-2005-08-3329 16291593

[126] Wang Z Li Y Kong D Sarkar FH The role of notch signaling pathway in epithelial-mesenchymal transition (EMT) during development and tumor aggressiveness. Curr Drug Targets (2010) 11:745–51 10.2174/138945010791170860 20041844 PMC3084452

[127] Sethi S Macoska J Chen W Sarkar FH Molecular signature of epithelial-mesenchymal transition (EMT) in human prostate cancer bone metastasis. Am J Transl Res (2010) 3:90–9 21139809 PMC2981429

[128] Leong KG Niessen K Kulic I Raouf A Eaves C Pollet I Jagged1-mediated notch activation induces epithelial-to-mesenchymal transition through slug-induced repression of E-cadherin. J Exp Med (2007) 204:2935–48 10.1084/jem.20071082 17984306 PMC2118507

[129] Brabletz S Bajdak K Meidhof S Burk U Niedermann G Firat E The ZEB1/miR-200 feedback loop controls notch signalling in cancer cells. EMBO J (2011) 30:770–82 10.1038/emboj.2010.349 21224848 PMC3041948

[130] Yang Y Ahn Y-H Gibbons DL Zang Y Lin W Thilaganathan N The notch ligand Jagged2 promotes lung adenocarcinoma metastasis through a miR-200-dependent pathway in mice. J Clin Invest (2011) 121:1373–85 10.1172/JCI42579 21403400 PMC3069760

[131] Hassan KA Wang L Korkaya H Chen G Maillard I Beer DG Notch pathway activity identifies cells with cancer stem cell-like properties and correlates with worse survival in lung adenocarcinoma. Clin Cancer Res (2013) 19:1972–80 10.1158/1078-0432.CCR-12-0370 23444212 PMC3630232

[132] Apostolou P Toloudi M Ioannou E Kourtidou E Chatziioannou M Kopic A Study of the interaction among notch pathway receptors, correlation with stemness, as well as their interaction with CD44, dipeptidyl peptidase-IV, hepatocyte growth factor receptor and the SETMAR transferase, in colon cancer stem cells. J Recept Signal Transduct Res (2013) 33:353–8 10.3109/10799893.2013.828072 23964856

[133] Zhang X-P Zheng G Zou L Liu H-L Hou L-H Zhou P Notch activation promotes cell proliferation and the formation of neural stem cell-like colonies in human glioma cells. Mol Cell Biochem (2008) 307:101–8 10.1007/s11010-007-9589-0 17849174

[134] Mazzone M Selfors LM Albeck J Overholtzer M Sale S Carroll DL Dose-dependent induction of distinct phenotypic responses to notch pathway activation in mammary epithelial cells. Proc Natl Acad Sci U S A (2010) 107:5012–7 10.1073/pnas.1000896107 20194747 PMC2841923

[135] Eliasz S Liang S Chen Y De Marco MA Machek O Skucha S Notch-1 stimulates survival of lung adenocarcinoma cells during hypoxia by activating the IGF-1R pathway. Oncogene (2010) 29:2488–98 10.1038/onc.2010.7 20154720 PMC2861728

[136] Hsu H-J Drummond-Barbosa D Insulin signals control the competence of the *Drosophila* female germline stem cell niche to respond to notch ligands. Dev Biol (2011) 350:290–300 10.1016/j.ydbio.2010.11.032 21145317

[137] Ouellet J Li S Roy R Notch signalling is required for both dauer maintenance and recovery in *C. elegans*. Development (2008) 135:2583–92 10.1242/dev.012435 18599512

[138] McMahon AP Ingham PW Tabin CJ Developmental roles and clinical significance of hedgehog signaling. Curr Top Dev Biol (2003) 53:1–114 12509125 10.1016/s0070-2153(03)53002-2

[139] Milla LA González-Ramírez CN Palma V Sonic hedgehog in cancer stem cells: a novel link with autophagy. Biol Res (2012) 45:223–30 10.4067/S0716-97602012000300004 23283432

[140] Sanchez P Hernández AM Stecca B Kahler AJ DeGueme AM Barrett A Inhibition of prostate cancer proliferation by interference with SONIC HEDGEHOG-GLI1 signaling. Proc Natl Acad Sci U S A (2004) 101:12561–6 10.1073/pnas.0404956101 15314219 PMC514658

[141] Athar M Tang X Lee JL Kopelovich L Kim AL Hedgehog signalling in skin development and cancer. Exp Dermatol (2006) 15:667–77 10.1111/j.1600-0625.2006.00473.x 16881963

[142] Yang Z-J Ellis T Markant SL Read T-A Kessler JD Bourboulas M Medulloblastoma can be initiated by deletion of patched in lineage-restricted progenitors or stem cells. Cancer Cell (2008) 14:135–45 10.1016/j.ccr.2008.07.003 18691548 PMC2538687

[143] Katoh Y Katoh M Hedgehog signaling pathway and gastrointestinal stem cell signaling network (review). Int J Mol Med (2006) 18:1019–23 17089004

[144] Maitah MY Ali S Ahmad A Gadgeel S Sarkar FH Up-regulation of sonic hedgehog contributes to TGF-β1-induced epithelial to mesenchymal transition in NSCLC cells. PLoS One (2011) 6:e16068 10.1371/journal.pone.0016068 21249152 PMC3020967

[145] Dahmane N Sánchez P Gitton Y Palma V Sun T Beyna M The sonic hedgehog-Gli pathway regulates dorsal brain growth and tumorigenesis. Development (2001) 128:5201–12 11748155 10.1242/dev.128.24.5201

[146] Ruiz i Altaba A Stecca B Sánchez P Hedgehog – Gli signaling in brain tumors: stem cells and paradevelopmental programs in cancer. Cancer Lett (2004) 204:145–57 10.1016/S0304-3835(03)00451-8 15013214

[147] Ahlfeld J Favaro R Pagella P Kretzschmar HA Nicolis S Schüller U Sox2 requirement in sonic hedgehog-associated medulloblastoma. Cancer Res (2013) 73:3796–807 10.1158/0008-5472.CAN-13-0238 23596255

[148] Takanaga H Tsuchida-Straeten N Nishide K Watanabe A Aburatani H Kondo T Gli2 is a novel regulator of sox2 expression in telencephalic neuroepithelial cells. Stem Cells (2009) 27:165–74 10.1634/stemcells.2008-0580 18927476

[149] Clement V Sanchez P de Tribolet N Radovanovic I Ruiz i Altaba A HEDGEHOG-GLI1 signaling regulates human glioma growth, cancer stem cell self-renewal, and tumorigenicity. Curr Biol (2007) 17:165–72 10.1016/j.cub.2006.11.033 17196391 PMC1855204

[150] Rao G Pedone CA Del Valle L Reiss K Holland EC Fults DW Sonic hedgehog and insulin-like growth factor signaling synergize to induce medulloblastoma formation from nestin-expressing neural progenitors in mice. Oncogene (2004) 23:6156–62 10.1038/sj.onc.1207818 15195141

[151] Hahn H Wojnowski L Specht K Kappler R Calzada-Wack J Potter D Patched target Igf2 is indispensable for the formation of medulloblastoma and rhabdomyosarcoma. J Biol Chem (2000) 275:28341–4 10.1074/jbc.C000352200 10884376

[152] Fernandez C Tatard VM Bertrand N Dahmane N Differential modulation of Sonic-hedgehog-induced cerebellar granule cell precursor proliferation by the IGF signaling network. Dev Neurosci (2010) 32:59–70 10.1159/000274458 20389077 PMC2866582

[153] Corcoran RB Bachar Raveh T Barakat MT Lee EY Scott MP Insulin-like growth factor 2 is required for progression to advanced medulloblastoma in patched1 heterozygous mice. Cancer Res (2008) 68:8788–95 10.1158/0008-5472.CAN-08-2135 18974121 PMC2597356

[154] Hsieh A Ellsworth R Hsieh D Hedgehog/GLI1 regulates IGF dependent malignant behaviors in glioma stem cells. J Cell Physiol (2011) 226:1118–27 10.1002/jcp.22433 20857406

[155] Parathath SR Mainwaring LA Fernandez-L A Campbell DO Kenney AM Insulin receptor substrate 1 is an effector of sonic hedgehog mitogenic signaling in cerebellar neural precursors. Development (2008) 135:3291–300 10.1242/dev.022871 18755774 PMC2673703

[156] Kenney AM Widlund HR Rowitch DH Hedgehog and PI-3 kinase signaling converge on Nmyc1 to promote cell cycle progression in cerebellar neuronal precursors. Development (2004) 131:217–28 10.1242/dev.00891 14660435

[157] Browd SR Kenney AM Gottfried ON Yoon JW Walterhouse D Pedone CA N-myc can substitute for insulin-like growth factor signaling in a mouse model of sonic hedgehog-induced medulloblastoma. Cancer Res (2006) 66:2666–72 10.1158/0008-5472.CAN-05-2198 16510586

[158] Hartmann W Koch A Brune H Waha A Schüller U Dani I Insulin-like growth factor II is involved in the proliferation control of medulloblastoma and its cerebellar precursor cells. Am J Pathol (2005) 166:1153–62 10.1016/S0002-9440(10)62335-8 15793295 PMC1602379

[159] Werner H Shalita-Chesner M Abramovitch S Idelman G Shaharabani-Gargir L Glaser T Regulation of the insulin-like growth factor-I receptor gene by oncogenes and antioncogenes: implications in human cancer. Mol Genet Metab (2000) 71:315–20 10.1006/mgme.2000.3044 11001824

